# The SLC25 Carrier Family: Important Transport Proteins in Mitochondrial Physiology and Pathology

**DOI:** 10.1152/physiol.00009.2020

**Published:** 2020-09-01

**Authors:** Edmund R.S. Kunji, Martin S. King, Jonathan J. Ruprecht, Chancievan Thangaratnarajah

**Affiliations:** 1 Medical Research Council Mitochondrial Biology Unit, University of Cambridge, Cambridge, United Kingdom; 2 Groningen Biomolecular Sciences and Biotechnology Institute, Membrane Enzymology, University of Groningen, Groningen, The Netherlands

**Keywords:** mitochondrial physiology, mitochondrial disease, impaired transport mechanism, pathological mutations, bioenergetics

## Abstract

Members of the mitochondrial carrier family (SLC25) transport a variety of compounds across the inner membrane of mitochondria. These transport steps provide building blocks for the cell and link the pathways of the mitochondrial matrix and cytosol. An increasing number of diseases and pathologies has been associated with their dysfunction. In this review, the molecular basis of these diseases is explained based on our current understanding of their transport mechanism.

## Introduction

**Figure 1:**
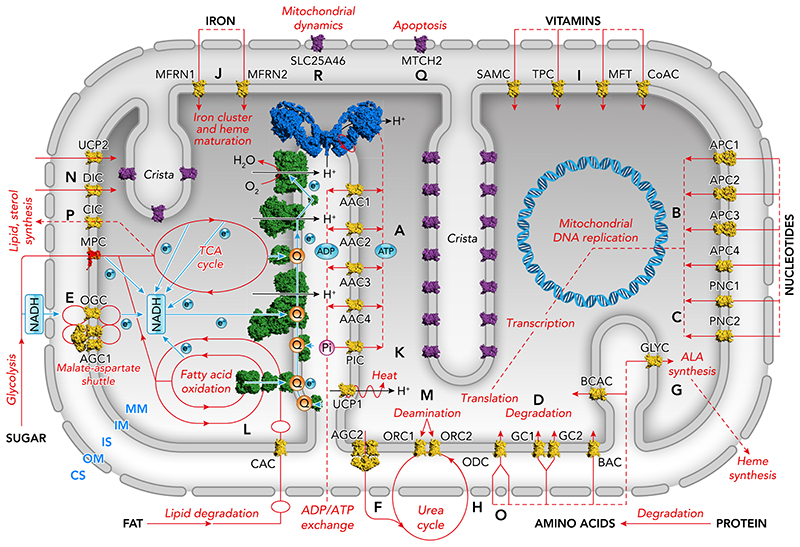
The role of the human mitochondrial carrier family (SLC25) in metabolism and mitochondrial function Schematic representation of the mammalian mitochondrion. Shown in green are the electron transfer chain complexes. *Bottom* to *top:* glycerophosphate dehydrogenase, fatty acid-dehydrogenase-electron transfer flavoprotein, dihydroorotate dehydrogenase, and complex I to IV with cytochrome c. Shown in blue is the dimer of ATP synthase, and in red/orange the mitochondrial pyruvate carrier heterodimer (MPC). Shown in purple and yellow are unidentified and identified mitochondrial carriers, respectively. AAC1–4, ADP/ATP carriers; AGC1–2, aspartate/glutamate carriers; APC1–4, ATP-Mg/Pi carriers; BAC, basic amino acid carrier; CAC, carnitine-acylcarnitine carrier; CIC, citrate carrier; DIC, dicarboxylate carrier; GC1–2, glutamate carriers; GLYC, glycine carrier; MTFRN1–2, mitoferrins; ODC, oxoadipate carrier; OGC, oxoglutarate carrier; ORC1–2, ornithine carriers; PIC, phosphate carrier; SAMC, *S*-adenosylmethionine carrier; TPC, thiamine pyrophosphate carrier; UCP1, uncoupling protein; UCP2, uncoupling-like protein 2; CS, cytosol; IS, intermembrane space; OM, outer membrane; IM, inner membrane; MM, mitochondrial matrix; Q, ubiquinone; Pi, phosphate; ALA, aminolevulinic acid.

The inner membrane of mitochondria is highly impermeable to molecules and ions, which is a key property required for energy conversion in oxidative phosphorylation. Therefore, a large number of transport proteins and channels are required to transport molecules and ions across this membrane to link cytosolic and mitochondrial metabolism, and to provide compounds for building and maintenance of the mitochondrion and the cell. In fact, all major food groups pass through the mitochondrion as part of central metabolism, including the degradation products of fats, sugars, and proteins, as well as nucleotides, vitamins, and inorganic ions (FIGURE 1). Most of the transport steps are carried out by members of the mitochondrial carrier family (SLC25), the topic of this review, but there are also other transporter families, such as the mitochondrial pyruvate carrier (SLC54) (19, 61), sideroflexins (SLC56) (87, 127, 170), and mitochondrial ABC transporters (98, 148). Mitochondrial carriers provide key transport steps in a variety of metabolic pathways, such as the oxidation of degradation products of fats and sugars, the degradation, synthesis, and interconversion of amino acids, and the synthesis of iron sulfur clusters and heme, but also in ion homeostasis, mitochondrial macromolecular synthesis, heat production, mitochondrial dynamics, signaling, cellular differentiation, development, and cell death (89, 123). The genes of 53 mitochondrial carriers of the SLC25 family have been identified in the human genome (89, 123), based on their shared sequence features (137, 147) and structural properties (128, 141, 143, 144). Most carriers are found in the inner membrane of the mitochondrion, but an adenine nucleotide transporter (SLC25A17) has been located in the peroxisome, where it supplies ATP for energy-requiring processes (161). Moreover, two highly divergent carriers, SLC25A46 and SLC25A50 (MTCH2), have been localized to the outer membrane of the mitochondrion (2, 172), where they might have some involvement in mitochondrial dynamics and apoptosis, respectively, but no transport function has been assigned to them.

Mitochondrial carriers are functional as monomers (10–12, 32, 91). The only confirmed exception is the mitochondrial aspartate/glutamate carrier, which is a structural dimer through the interactions of the NH_2_-terminal calcium regulatory domains, but the carrier domains, which are involved in transport, function separately (see further below) (157). Recently, a claim was made for the bovine mitochondrial ADP/ATP carrier being monomeric and dimeric using native mass spectrometry of intact membranes (28), but the reported mass deviates significantly from those observed by others (7, 62, 153). To account for the difference, the carrier was proposed to have an unusually large number of modifications, which have not been observed in other studies (7, 153) or in the structure (128). Furthermore, the species observed by native mass spectrometry lacks three tightly bound cardiolipin molecules (28), which are observed consistently in other studies (10, 14, 96, 128, 141, 142), and thus does not represent the native ADP/ATP carrier.

Given the central role of mitochondrial carriers in cellular metabolism and physiology, it is not surprising that mutations in mitochondrial carriers have been associated with a large number of pathologies. Due to recent advances in sequencing technologies, this number is likely to rise rapidly, as new links to disease are discovered frequently, including for mitochondrial carriers that have no assigned function yet. Although originally thought to be relatively rare, it is now clear that pathologies involving mitochondrial carriers may be among the most prevalent of all mitochondrial diseases. For example, citrin deficiency, which is caused by disease variants of the mitochondrial aspartate/glutamate carrier (SLC25A13), has a very high frequency in Far Eastern populations (146). Current estimates, based on pathogenic allele frequencies, are 1:17,000 in Japan and China, 1:9,000 in Taiwan, and 1:50,000 in Korea, but the disease has now also been discovered in other populations, making it panethnic (38, 122). With few exceptions most pathologies are inherited in an autosomal recessive manner. The phenotypic manifestation of these pathologies is highly variable, starting at different ages, affecting various organs in different ways, and is dependent on the type of mutation, i.e., deletion, missense, nonsense, inversion, or splice-site mutation. The pathologies can be broadly divided into developmental, metabolic, and neuromuscular diseases. Understanding the molecular basis for these diseases is vital for diagnosis and prognosis of the disease, and for development of effective treatments and therapies.

In this review, we provide a comprehensive overview of the role of mitochondrial carriers in physiology and pathology. We describe all pathogenic missense mutations that have been identified to date in the context of new insights into the structural mechanism of these transport proteins. This analysis can be used to predict with better accuracy which mutations are likely to be pathological, resulting in a dysfunctional mitochondrial carrier. The data presented in this review may also help to identify new pathological variants and associated mitochondrial diseases.

## The Role of Mitochondrial Carriers in Physiology and Pathology

With its 53 members, the mitochondrial carrier family SLC25 is the largest solute transporter family in humans. Here, we first briefly review the function of the known human mitochondrial carriers in cellular physiology and pathology, but for more detailed information the reader is referred to Ref. 124.

### Nucleotide Transport

**Figure 2:**
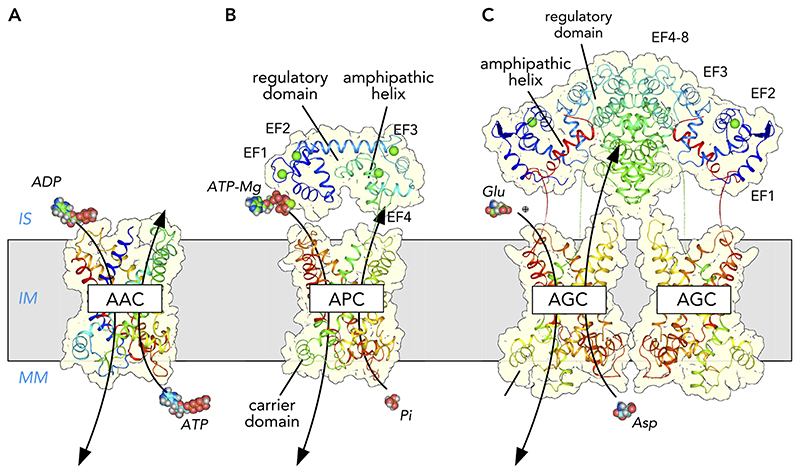
Structures of the mitochondrial ADP/ATP carrier and the calcium-regulated ATP-Mg/phosphate carrier and aspartate/glutamate carrier Structures of three different mitochondrial carriers, based on PDB entries 1OKC (128) and 4C9Q (141) for the carrier domains and 4P5W (157) and 4ZCU (59) for the calcium-regulatory domains. *A:* the mitochondrial ADP/ATP carrier. *B:* the mitochondrial ATP-Mg/Pi carrier consists of three domains: *1*) NH_2_-terminal calcium-regulatory domain with four EF-hands (EF1–4), each binding calcium, *2*) amphipathic helix, and *3*) COOH-terminal carrier domain. *C:* the aspartate/glutamate carrier also has a three-domain structure, but with a different order: *1*) NH_2_-terminal calcium-regulatory domain, *2*) carrier domain, and *3*) COOH-terminal amphipathic helix. The NH_2_-terminal domain has eight EF-hand folds, but only EF-hand 2 is capable of binding calcium, which together with EF-hands 1 and 3 forms a calcium-responsive mobile unit. EF-hands 4–8 have evolved to form a static dimerization interface. The structures are shown in a cartoon representation, colored from the NH_2_-terminus in blue to the COOH-terminus in red. Also shown are the canonical substrates as well as calcium ions (green), magnesium ion (chartreuse), and protons (white) in sphere representations. IS, intermembrane space; OM, outer membrane; IM, inner membrane; MM, mitochondrial matrix.

Mitochondria require nucleotides for a host of important functions, such as the synthesis of ATP, mitochondrial RNA and DNA synthesis, enzymatic reactions, and regulation. The best-known member of the SLC25 family is the mitochondrial ADP/ATP carrier, also called adenine nucleotide translocase or translocator (ANT). The carrier imports ADP into the mitochondrial matrix, where it is converted to ATP by ATP synthase, and exports the newly synthesized ATP to the cytosol, where it fuels the metabolic energy-requiring processes that are vital for cell survival (7, 83, 90, 144). There are four different paralogs in humans, AAC1, AAC2, AAC3, and AAC4 (SLC25A4, SLC25A5, SLC25A6, and SLC25A31, respectively), which are expressed in a tissue-dependent manner (FIGURE 1A) (31, 40, 88, 115). The mitochondrial ADP/ATP carriers carry out equimolar exchange of ADP and ATP, and thus do not alter the total adenine nucleotide pool in the mitochondrial matrix. The mitochondrial ADP/ATP carrier has been extensively characterized structurally (FIGURE 2A) (128, 141–144). In the absence of adenine nucleotides and in the presence of fatty acids, the mitochondrial ADP/ATP carrier may function as an uncoupling protein, transporting protons (13, 20), but a molecular mechanism is lacking.

The human mitochondrial ADP/ATP carrier AAC1 (SLC25A4) has three associated pathologies, which are quite different in nature: *1*) late-onset dominant progressive external ophthalmoplegia with mitochondrial DNA deletions (OMIM 609283) (77), *2*) recessive mitochondrial DNA depletion syndrome (OMIM 615418) (53, 125), and *3*) early onset dominant de novo mutations leading to mitochondrial disease, varying from lethal to mild severity (OMIM 103220) (81, 158). The complexity of the genetics underlying these three diseases has not been fully explained. As will be shown below, the carrier has all the features required for its transporter function, so the dominant inheritance cannot be explained by dimerization, as was thought previously (91). This notion is supported further by the fact that the early onset dominant disease mutations involve key functional residues on the inside of the carrier, which cannot be involved in dimerization (158). Every day, the ADP/ATP carriers transport approximately our own body weight in ADP and ATP across the inner membrane of mitochondria, meaning that the dominant inheritance could well be explained by haplo-insufficiency. In agreement, the early onset de novo dominant mutations affect highly conserved residues that are central to the transport mechanism (p.Lys33Gln, p.Arg80His, and p.Arg235Gly), whereas late-onset domain mutations affect non-conserved residues on the periphery of the protein without an obvious role in the mechanism (e.g., p.Ala90Asp, p.Asp104Gly, Leu98Pro, and Ala114Pro). Not fitting this genetic explanation are the recessive mutations, which lead to mitochondrial DNA deletions and mitochondrial disease only in homozygotes, whereas the heterozygotes are unaffected (53), but they may give rise to biogenesis rather than functional issues (e.g., p.Ala123Asp, p.Arg263Pro). However, it would be impossible for humans to live without functional ADP/ATP carriers, since cells would be reliant on glycolysis and fermentation alone, providing low yields of ATP. The oxidative phosphorylation of sugars, fats, and amino acids in mitochondria is critically dependent on ADP/ATP carriers. This notion is supported by the fact that the powerful inhibitors carboxyatractyloside and bongkrekic acid kill humans, showing that human life cannot sustain itself without mitochondrial ADP/ATP transport. There are three other paralogs of the mitochondrial ADP/ATP carrier, AAC2, AAC3, and AAC4, which are expressed at different levels and/or may be upregulated to compensate for the missing transport activity in homozygous recessive patients. In addition, there are the four paralogs of the ATP-Mg/Pi carrier and some vitamin transporters, such as the CoA transporter (SLC25A42) (51) and thiamine pyrophosphate carrier (SLC25A19) (39), which can also transport adenine nucleotides in addition to their key substrates. The complexity of adenine nucleotide transport might explain viability in the homozygous-recessive cases, and the variation in severity and onset of the pathogenicity in the heterozygous-dominant cases (81, 158). The mitochondrial ADP/ATP carrier can also transport the deoxy forms of ADP and ATP, which are required for mitochondrial DNA replication, which could explain the role of disease variants in mitochondrial DNA deletions and maintenance, aside from the production of ATP. Finally, very little is known about the effect of these mutations on the biogenesis of the disease variants. Therefore, other genetic mechanisms, such as gain of function through aggregation, are also pursued as an explanation (100, 101). However, it is clear that this important issue is unresolved, requiring a more complete picture of adenine nucleotide transport in mitochondria.

The net import and export of adenine nucleotides is carried out by mitochondrial ATP-Mg/Pi carriers, which exchange phosphate for adenine nucleotides, coupled to magnesium or protons, in an electroneutral way (8, 36, 45). This transport activity allows the mitochondrion to respond to changes in energetic demand and to replenish adenine nucleotide pools after mitochondrial division and macromolecular synthesis (8, 26, 45). There are three human paralogs, APC1 (SLC25A24), APC2 (SLC25A23), and APC3 (SLC25A25), which are calcium-regulated (5, 45), and a fourth paralog, APC4 (SLC25A41), which is not (160) (FIGURE 1B). APC1–3 have three domains: *1*) an NH_2_-terminal calcium-regulatory domain with four calcium-binding EF-hands, *2*) an amphipathic helix, and *3*) a COOH-terminal carrier domain (59) (FIGURE 2B). In the current model of calcium regulation, the amphipathic helix is bound to the regulatory domain in the presence of calcium, allowing transport by the carrier domain to occur (59, 169). In the absence of calcium, the amphipathic helix is released from the regulatory domain and binds to the carrier domain, leading to inhibition of transport (58, 60). APC4 lacks both the NH_2_-terminal calcium-regulatory domain and the amphipathic helix (160), which accounts for the absence of calcium regulation.

Dysfunction of the mitochondrial Mg-ATP/phosphate carrier APC1 (SLC25A24) leads to Fontaine Progeroid Syndrome, also described as Gorlin-Chaudhry-Moss or Fontaine-Farriaux syndrome (43, 138, 145, 168) (OMIM 612289). The mutations are spontaneous rather than inherited and lead to a developmental disease, which is characterized by prenatal and postnatal growth retardation, failure to thrive, a lack of subcutaneous adipose tissue, premature closure of certain skull bones (coronal craniosynostosis), and short distal phalanges of the fingers and toes. Other clinical features include abnormal hair patterns, skin agenesis, umbilical hernia, and progeroid facial appearance. APC1 variants are expressed in mitochondria, and they affect both the mitochondrial morphology and cell viability. There is also a decrease in mitochondrial ATP synthesis in fibroblasts, but only under stress conditions (168). The disease variants have not been characterized with respect to folding and transport function. Interestingly, the paralog APC3 (SLC25A25) has been implicated in left-right determination during development and is regulated by TRPP2 ion channels (64), showing that the Mg-ATP/phosphate carriers are also playing an important role in development.

There are two pyrimidine nucleotide carriers in humans (PNC1, SLC25A33 and PNC2, SLC25A36), which are required for mitochondrial DNA and RNA synthesis and breakdown (37, 52) (FIGURE 1C). SLC25A33 transports uracil, thymine, and cytosine (deoxy)nucleoside di- and tri-phosphates by an antiport mechanism, whereas SLC25A36 translocates both cytosine and uracil (deoxy)nucleoside mono-, di-, and tri-phosphates using a uniport or antiport mechanism (37, 52). Both carriers also transport guanine nucleotides, but not adenine (deoxy)-nucleotides. It has also been claimed that the pyrimidine carriers are involved in the uptake of zinc ions (85), even though several dedicated zinc transporters are likely to exist in mitochondria. In yeast, a separate mitochondrial GTP/GDP carrier has been identified (162), but an equivalent transporter has not been identified in humans. The most closely related carriers by sequence comparison are SLC25A51 and SLC25A52 (137), but no experimental data are available to support this notion. So far, no disease variants of PNC1 and 2 have been identified.

### Amino Acid Transport

Amino acids need to be transported into mitochondria for mitochondrial protein synthesis and for amino acid interconversion and degradation, which can generate metabolic energy. Several mitochondrial amino acid transporters have been identified, but some are still missing, importantly those for tryptophan, tyrosine, phenylalanine, methionine, glutamine, asparagine, and cysteine (FIGURE 1D).

The mitochondrial aspartate/glutamate carriers import glutamate and a proton, and they export aspartate from the mitochondrial matrix (9, 16, 18). AGC1 (SCL25A12, aralar) and AGC2 (SLC25A13, citrin) are expressed in excitable and non-excitable tissues, respectively, and play important roles in the malate-aspartate shuttle (FIGURE 1E), gluconeogenesis, purine and pyrimidine synthesis, the urea cycle (AGC2) (FIGURE 1F), and myelin synthesis (AGC1) (126). They have an unusual three-domain structure, consisting of *1)* a NH_2_-terminal calcium-regulatory domain, *2)* a carrier domain, and *3)* a COOH-terminal amphipathic helix (157) (FIGURE 2C). The NH_2_-terminal domain has eight EF-hand domains, but only EF-hand 2 is capable of binding calcium, whereas EF-hands 4–8 are involved in dimerization, generating a structural homo-dimer (FIGURE 2C). In the calcium-bound state, the amphipathic helix is bound to the regulatory domain, whereas in the absence of calcium the amphipathic helix is released (157), but the mechanism of regulation is not fully resolved.

Mutations of the gene coding for the liver paralog AGC2 (SLC25A13) causes neonatal and/or adult-onset type-II citrullinemia (OMIM 603859), an autosomal recessive disease characterized by hyperammonemia and citrullinemia, because of a dysfunctional urea cycle, as well as neuropsychiatric symptoms and fatty liver disease in later life (86, 122). Mutations of the gene coding for the brainspecific paralog AGC1 (SLC25A12) lead to early infantile epileptic encephalopathy due to hypomyelination (OMIM 603667). This is caused by the lack of mitochondrial aspartate export, which is required for the synthesis of *N*-acetyl-aspartate, a precursor for myelin synthesis (167).

Two related proteins, the mitochondrial glutamate carriers GC1 (SLC25A22) and GC2 (SLC25A18), are involved in the import of glutamate together with a proton but do not export aspartate (FIGURE 1D) (50). The glutamate carriers do not have the calcium-regulatory elements found in aspartate/glutamate carriers. Mutations in SLC25A22 (OMIM 609302) cause neonatal epileptic encephalopathy with suppression bursts (97, 110) or migrating partial seizures in infancy with poor developmental prognosis (129). The most likely reason for the disease phenotype is the role of mitochondrial glutamate transport in sustaining glutamate homeostasis in astrocytes (55).

The glycine carrier GLYC (SLC25A38) is involved in the import of glycine into mitochondria, where it reacts enzymatically with succinyl-CoA to form aminolevulinic acid (FIGURE 1G) (102). The gene was flagged because a disease variant leads to autosomal recessive sideroblastic anemia (OMIM 610819), caused by the inability of blood cells to synthesize heme due to defective glycine transport (48). Aminolevulinic acid is transported by an unknown transporter into the cytosol, where it is used as a precursor for porphyrin synthesis. The resulting coproporphyrinogen III is then transported into mitochondria by an unknown transporter for incorporation of iron and insertion into heme-containing proteins.

The ornithine carriers ORC1 and ORC2 (SLC25A15 and SLC25A2) catalyze the exchange of ornithine and citrulline, which links the fixation of ammonia in mitochondria to the urea cycle (FIGURE 1H) (47). The substrate specificity and binding of these two paralogs have been studied in detail (111). Dysfunction of ORC1 (SLC25A15) leads to HHH syndrome (OMIM 603861), characterized by hyperornithinemia, hyperammonemia, and homocitrullinuria, due to an impaired urea cycle, which is required for the deamination of amino acids (22, 65). ORC2 (SLC25A2) (OMIM 608157) may be responsible for the milder phenotype in HHH patients, secondary to a gene redundancy effect. The basic amino acid carrier BAC (SLC25A29) is closely related to ORC1 and 2, but transports arginine, lysine, homoarginine, methylarginine, and, to a much lesser extent, ornithine and histidine (131), and has thus far no pathology associated with it.

Recently, the mitochondrial carrier for branched-chain amino acids (SLC25A44) was identified (FIGURE 1D) (171). The branched-chain amino acids valine, leucine, and isoleucine can be degraded to provide metabolic energy and are required for the synthesis of proteins in mitochondria. The carrier was discovered in brown adipose tissue upon cold exposure, where branched-chain amino acids can be used as a fuel for thermogenesis.

### Vitamin Transport

Many vitamins need to be transported into mitochondria, where they serve as co-factors or as donors of key functional groups in the enzymatic reactions of the mitochondrial matrix (FIGURE 1I).

The mitochondrial *S*-adenosylmethionine carrier SAMC (SLC25A26) imports *S*-adenosylmethionine into mitochondria, which is required for methylation reactions of DNA, RNA, and protein in the mitochondrial matrix, and exports the product *S*-adenosylhomocysteine (3). A missense mutation in the gene coding for this protein causes intramitochondrial methylation deficiency (OMIM 611037) in agreement with this function, leading to oxidative phosphorylation deficiency (82).

The human thiamine pyrophosphate transporter TPC (SLC25A19) was initially identified as a deoxynucleotide transporter (39), but its main function is the transport of thiamine pyrophosphate, which is an important co-factor in dehydrogenase reactions (99). Defective transport of thiamine pyrophosphate is the cause of Amish microcephaly, which is characterized by profound congenital microcephaly, delayed psychomotor development, and lactic and alpha-ketoglutaric aciduria (OMIM 606521) (78). A second mutation was reported in siblings of non-Amish background (OMIM 613710), expanding the phenotypes associated with the *SLC25A19* gene. These patients showed recurrent episodes of flaccid paralysis and encephalopathy associated with bilateral striatal necrosis and chronic progressive polyneuropathy caused by a missense mutation in SLC25A19 (155).

Mitochondria require folate for one-carbon metabolism and flavins for electron transfer steps in the respiratory chain. The transport of folate and flavin have both been assigned to a single mitochondrial carrier (SLC25A32) (154, 159). The substrate binding site has the typical features of an adenine binding pocket, agreeing with the flavin assignment (94, 136, 137). When mutated, the disease variant of this carrier causes exercise intolerance (OMIM 610815), and riboflavin supplementation proved to be beneficial to the patients, indicating that the most likely substrate of this carrier is flavin rather than folate (149).

Many reactions in the mitochondrial matrix, such as dehydrogenase activities, require coenzyme A (CoA) as co-factor. Coenzyme A is synthesized outside of mitochondria and must be transported into the mitochondrial matrix. The CoA transporter (SLC25A42) was initially identified based on database searches and showed expression in all tissues, with the highest levels detected in adipose tissue, and high levels detected in hypothalamus and all brain coronal sections (57). The disease variant of the CoA transporter (OMIM 610823) causes mitochondrial myopathy with muscle weakness and lactic acidosis, whereas other tissues and cognitive functions are not impaired (150). Another report described variable clinical manifestations, including lactic acidosis, developmental regression, and epilepsy (4).

Before SLC25A42 was identified as the CoA transporter, this transport activity was putatively assigned to a protein encoded by the *SLC25A16* gene (133). Both SLC25A16 and SLC25A42 are phylogenetically related to the yeast protein Leu5p and are capable of complementing the yeast knockout strain (51). The function and kinetic parameters of SLC25A42 were determined in transport assays with substrate specificities restricted to CoA, dephospho-CoA, ADP, and adenosine 3′,5′-diphosphate (51). The human protein encoded by the *SLC25A16* gene has been identified through a possible association with a thyroid disease called Grave’s disease, but it has not been demonstrated that it can transport CoA (173). To date only a single homozygous mutation has been reported in the *SLC25A16* gene (OMIM 139080), causing a nail disorder of the hand with different severity levels of onychodystrophy (79), but a direct correlation to function has not been confirmed either.

### Inorganic Ion Transport

Inorganic ions also need to be transported into the mitochondrial matrix, where they function as cofactors, as substrates for enzymatic reactions, and as regulators. There are likely to be transporters of other families as well as channels involved in inorganic ion transport, but they are beyond the scope of this review.

The mitoferrins SLC25A37 (MFRN1) and SLC25A28 (MFRN2) have been proposed to transport iron ions into mitochondria for incorporation into heme and iron-sulfur cluster synthesis, as well as other functions (FIGURE 1J) (113, 151). MFRN1 is highly expressed in differentiating erythroid cells and in other tissues at low levels, whereas MFRN2 is expressed ubiquitously in non-erythroid tissues (29, 151). Abnormal MFRN1 expression might contribute to erythropoietic protoporphyria phenotype, in agreement with this notion (165).

The mitochondrial phosphate carrier PIC (SLC25A3) imports inorganic phosphate for the synthesis of ATP (140) together with a proton (FIGURE 1K) (95). There are two alternative splicing variants showing different kinetic parameters and different expression profiles: whereas isoform A is highly expressed in heart, skeletal muscle, diaphragm (49), and pancreas (67), isoform B is expressed in all tested tissues, i.e., lung, kidney, brain, thymus, liver, heart, skeletal muscle, and diaphragm (49), albeit at lower levels compared with isoform A (67). The phosphate carrier is fundamental in maintaining the inorganic phosphate pool in the mitochondrial matrix, but there are also other carriers capable of transporting phosphate, such as the dicarboxylate carrier DIC (SLC25A10) and the mitochondrial ATP-Mg/Pi carriers APC1 (SLC25A24), APC2 (SLC25A23), APC3 (SLC25A25), and APC4 (SLC25A41) (5, 45, 160). Phosphate carrier deficiency leads to lactic acidosis, hypertrophic cardiomyopathy, muscular hypotonia, and early mortality (OMIM 600370), in agreement with the notion that its transport activity is the main pathway for phosphate import for ATP synthesis (15, 105, 106).

### Fatty Acid Transport

The carnitine/acylcarnitine carrier CAC (SLC25A20) is a key component of the carnitine cycle and imports acyl-carnitine into mitochondria for fatty acid β-oxidation and exports carnitine (FIGURE 1L) (66, 70). Mutations cause carnitine/acylcarnitine carrier deficiency, an autosomal recessive disorder characterized by severe, neonatal onset with cardiomyopathy or a milder phenotype with hypoglycemia, but no cardiomyopathy (OMIM 613698) (66). The inability to transport fatty acid chains into mitochondria makes the patients dependent on carbohydrates and amino acids for energy metabolism.

### Uncoupling Protein

The uncoupling protein UCP1 (SLC25A7) is predominantly found in brown adipose tissue of neonatal mammals (6, 118, 120) but is also found in the supraclavicular and the neck regions in adults in later life (FIGURE 1M) (116). UCP1 dissipates the proton motive force, short-circuiting the mitochondrion, which leads to the production of heat. The generation of heat from the oxidation of brown adipose fat protects the newly born against cold stress of vital organs. UCP1 is activated by fatty acids and inhibited by purine nucleotides (134), but the mechanism is still debated (23, 33, 44, 72, 84, 119). UCP1 is monomeric and binds three cardiolipins and a single purine nucleotide (96). Based on sequence analysis, UCP1 has retained all of the key features of mitochondrial carriers, indicating that it operates by a conventional carrier-like mechanism (33, 96). The transport of protons induced by fatty acids is relatively slow (44), which would fit a transporter rather than a channel mechanism, but a molecular mechanism has not been resolved. Although there are other closely related proteins (see below), UCP1 is most likely the only one involved in thermogenesis. There are no known disease states associated with mutations in the gene coding for UCP1.

### Dicarboxylate Transport

Dicarboxylates (FIGURE 1N) need to be continuously exchanged across the mitochondrial inner membrane for many different pathways, such as the tricarboxylic acid cycle, malate/aspartate shuttle, gluconeogenesis, and amino acid metabolism.

The mitochondrial dicarboxylate carrier DIC (SLC25A10) is involved in the transport of malonate, malate, succinate, sulfate, thiosulphate, and phosphate by electroneutral exchange (46, 73). The carrier is involved in gluconeogenesis and ureogenesis, the metabolism of sulfur compounds, as well as de novo fatty acid synthesis (109). The mitochondrial oxoglutarate carrier OGC (SLC25A11) exchanges cytosolic malate for 2-oxoglutarate from the mitochondrial matrix and plays an important role in the malate-aspartate shuttle, the oxoglutarate-isocitrate shuttle, and gluconeogenesis (69). Mutations in the gene might be correlated to metastatic paragangliomas (21).

The oxodicarboxylate carrier ODC (SLC25A21) imports 2-oxoadipate and exports 2-oxoglutarate, playing a central role in the catabolism of lysine, hydroxylysine, and tryptophan (FIGURE 1O) (48). Oxodicarboxylate carrier deficiency (OMIM 607571) is associated with mitochondrial DNA depletion and spinal muscular atrophy-like disease, most likely caused by the accumulation of toxic amino acid breakdown products (17).

There are several closely related sequences to UCP1, such as UCP2 (SLC25A8), UCP3 (SLC25A9), UCP4 (SLC25A27), UCP5 (SLC25A14), and UCP6 (SC25A30), but they are likely to be transporters of carboxylic acids (54, 163), which is in agreement with their close phylogenetic relationship to dicarboxylate transporters (123) and with the properties of their substrate binding sites (137). Many unresolved questions remain with respect to their molecular properties and their role in thermogenesis and metabolism. There are also other potential dicarboxylate carriers, such as SLC25A34 and SLC25A35, but their role in metabolism has not been clarified.

### Tricarboxylate Transport

The tricarboxylate or citrate carrier (SLC25A1) catalyzes the electroneutral exchange of tricarboxylates (citrate, isocitrate) for another tricarboxylate, a dicarboxylate or phosphoenolpyruvate (68, 76, 103). An important physiological function is the export of citrate from the mitochondria to the cytosol for the production of acetyl CoA, which is a starting point for lipid, dolichol, ubiquinone, and sterol synthesis (see references in Ref. 103), and acetylation reactions (112) (FIGURE 1P). Citrate carrier deficiency (OMIM 190315), which is hallmarked by combined D-2- and L-2-hydroxyglutaric aciduria, leads to neonatal-onset epileptic encephalopathy with severe muscular weakness, respiratory distress, and lack of psychomotor development resulting in early death (30, 42, 114, 121, 132, 152), which is most likely due to the severe biosynthetic deficiencies (103). Many of the missense mutations have been characterized with respect to the function of SLC25A1 (27, 42, 103, 130).

### Apoptosis

SLC25A50 (MTCH2) is a partially characterized mitochondrial carrier, which acts as a receptor-like protein for the truncated BH3-interacting domain death agonist protein in the outer membrane of mitochondria, as part of the apoptosis pathway (FIGURE 1Q) (172). MTCH2 is likely to have a similar topology as other mitochondrial carriers (135), but unusually it is found in the mitochondrial outer membrane. A transported substrate has not yet been identified, if this protein has a transporter function at all, but MTCH2 was subsequently found to be required and sufficient for lipid homeostasis shifts (139).

### Mitochondrial Dynamics

The partially characterized mitochondrial carrier SLC25A46 is most likely involved in mitochondrial dynamics (FIGURE 1R). Overexpression leads to mitochondrial fragmentation, whereas knockdown results in hyperfilamentous mitochondria and mitochondrial hyperfusion, likely resulting from decreased fission. Loss of SLC25A46 was not associated with changes in total ATP concentration, mitochondrial DNA content, or membrane potential (2). SLC25A46 could interact with proteins associated with mitochondrial dynamics, such as OPA1 and MFN2, and with components of the mitochondrial contact site and mitochondrial cristae organizing system complex, which plays a role in cristae maintenance (71). SLC25A46 might also interact with components of an endoplasmic reticulum membrane protein complex involved in lipid transfer to mitochondria, which are also required for cristae growth and maintenance. SLC25A46 is found in the outer membrane of mitochondria (71), which is atypical for mitochondrial carriers except for MTCH2 (see above). It is possible that the carrier has evolved away from the canonical transporter function, since, so far, no substrates have been identified. Disease variants of SLC25A46 (OMIM 610826) lead to hereditary motor and sensory neuropathy type VIB (2) and in severe cases to death in infancy (1, 164).

### Uncharacterized Mitochondrial Carriers

The functions of a large number of mitochondrial carriers have not yet been assigned, limiting our understanding of their role in human physiology and pathology. Among them are SLC25A34, SLC25A35, SLC25A39, SLC25A40, SLC25A43, SLC25A45, SLC25A47, SLC25A48, SLC25A49 (MTCH1), SLC25A51 (MCART1), SLC25A52 (MCART2), and SLC25A53 (MCART6), which is roughly a quarter of the total. No substrates have been identified for SLC25A46 and SLC25A50 (MTCH2) (see above), although they have a role in mitochondrial dynamics and apoptosis, respectively. There is still a lot of debate on the role of UCP2 (SLC25A8), UCP3 (SLC25A9), UCP4 (SLC25A27), UCP5 (SLC25A14), and UCP6 (SLC25A30) in human physiology. In some other cases, the function of particular carriers has been disputed, for example SLC25A32, which has been described as a folate or flavin transporter. Counting these, the number of carriers that are not yet fully characterized is closer to a third of the total.

## The Molecular Basis of Pathogenic Missense Mutations

Missense mutations in 16 different carriers lead to human disease, but it is likely that this number will increase substantially as more links are discovered through genome and exome sequencing. These mutations can impair the structure and mechanism of the carrier, but can also cause issues with biogenesis, i.e., the expression, targeting, insertion, and folding of the disease variant. The impact of the vast majority of these missense mutations on the structure, function, and biogenesis of different carrier has not been studied experimentally. To discriminate between these different scenarios, it is important to understand the transport mechanism in detail. Recently, good progress has been made facilitating this assessment, although many details still need to be worked out. To explain the molecular impact of the pathogenic mutations on the function of mitochondrial carriers, it is important to explain their basic structure and transport mechanism first.

### Structures of Mitochondrial Carriers

**Figure 3:**
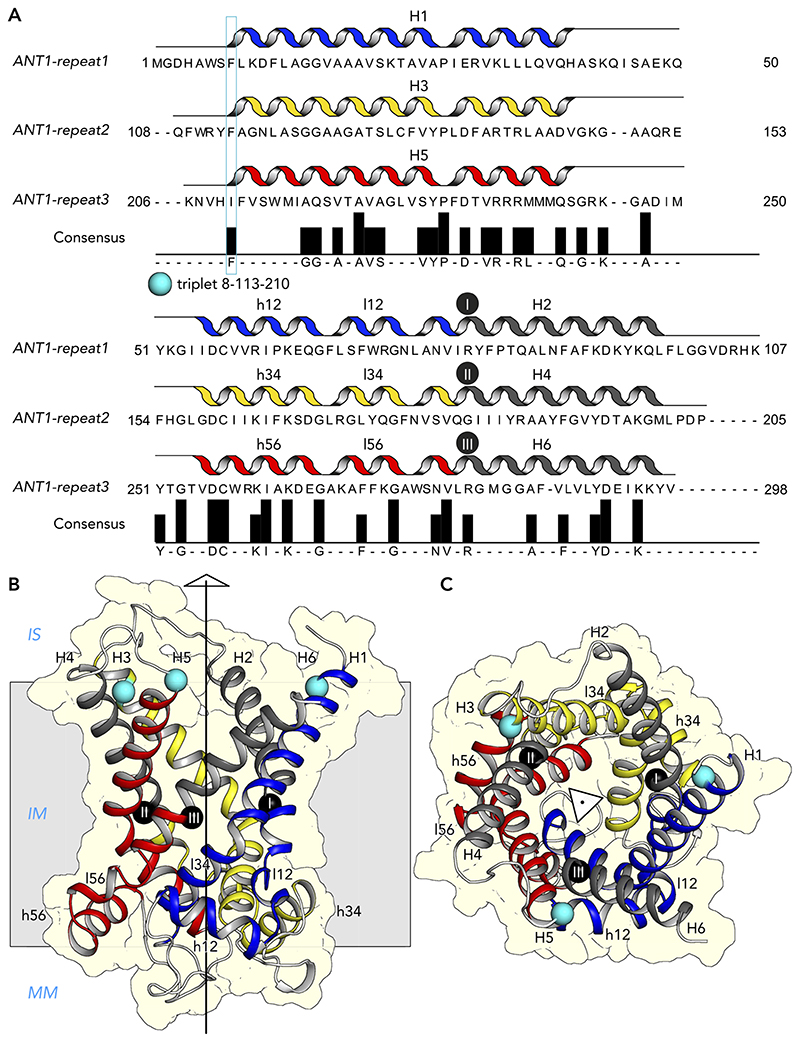
Mitochondrial carriers have a threefold pseudo-symmetrical structure *A:* aligned amino acid sequences of the three repeats of the human ADP/ATP carrier AAC1 (ANT1). Symmetrically conserved residues are shown in the consensus sequence and as bars, when present in at least two out of three repeats. *B* and *C:* comparative model of the human ADP/ATP carrier, based on PDB:1OKC (128), viewed from the membrane and the intermembrane space, respectively. Shown also is the threefold pseudosymmetrical axis, symbolized by an equilateral triangle. Odd-numbered (H1, H3, H5), matrix (h12, h34, h56), linker (l12, l34, l56), and even-numbered helices (H2, H4, H6) are shown in primary colors for the core elements and in gray for the gate elements (142). The black spheres with roman numerals show the positions of the three contact points of the substrate binding site (94, 136). The example, triplet 8–113–210, is indicated by a rectangle across the three repeats in *A* and by cyan spheres in *B*. IS, intermembrane space; IM, inner membrane; MM, mitochondrial matrix.

**Figure 4:**
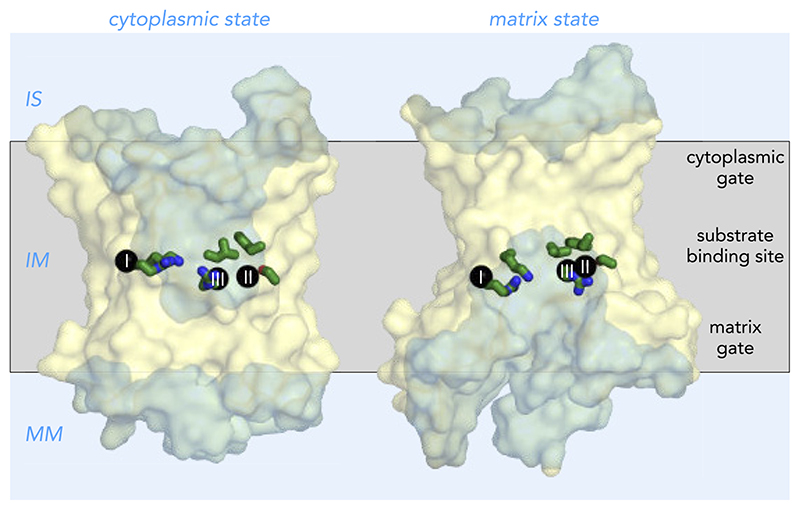
Alternating access transport mechanism for the mitochondrial ADP/ATP carrier Lateral view from the membrane of the mitochondrial ADP/ATP carrier in the cytoplasmic state (*left*) and matrix state (*right*). Shown are the cytoplasmic state of the bovine ADP/ATP carrier (PDB:1OKC) (128) and the matrix state of the ADP/ATP carrier of *Thermothelomyces thermophila* (PDB:6GCI) (142). The water-accessible surfaces are shown in light blue. Also indicated are the three main functional features: cytoplasmic gate, substrate binding site, and matrix gate. The black spheres with roman numerals are the contact points of the substrate binding site (94, 136). Shown in green are residues that most likely bind the adenine nucleotide substrates. IS, intermembrane space; IM, inner membrane; MM, mitochondrial matrix.

**Figure 5:**
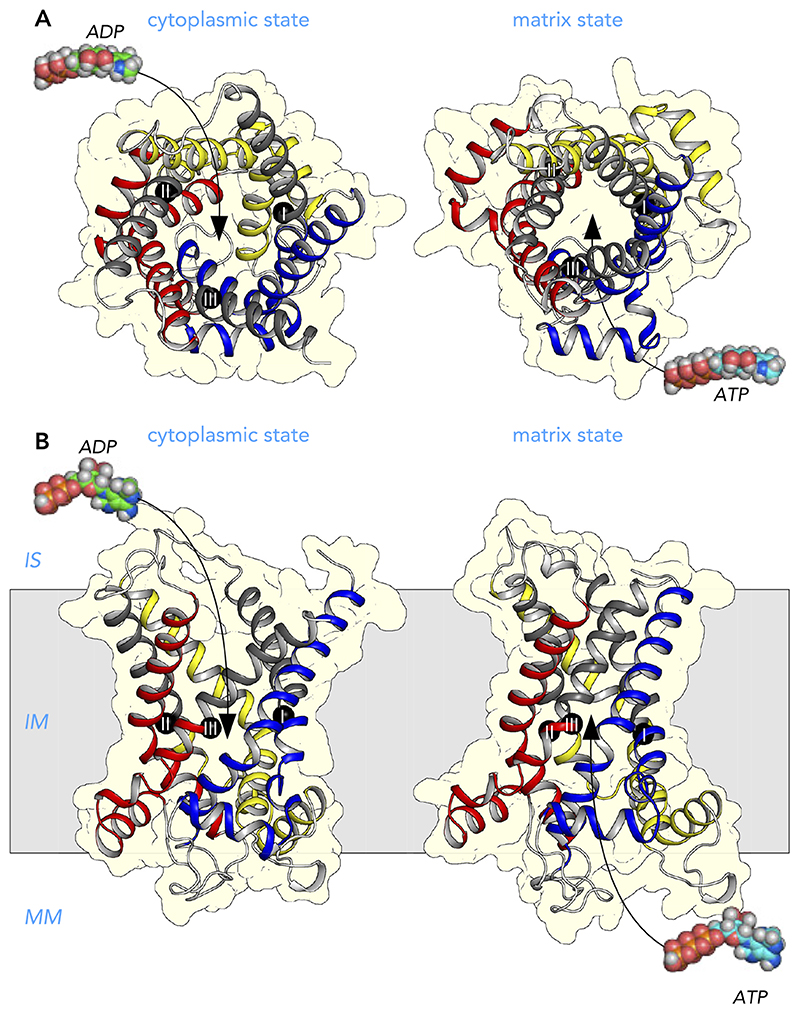
Structural changes in the transport cycle of the human mitochondrial ADP/ATP carrier View from the intermembrane space (*A*) and lateral view from the membrane of the human ADP/ATP carrier (*B*) in the cytoplasmic state (*left*) and matrix state (*right*). For the import of ADP, conformational changes involve the simultaneous outward rotation of the core elements, shown in primary colors, and inward rotation of the gate elements, shown in gray, in a coordinated way (142). For the export of ATP, the converse happens. Substrate binding increases the probability of the state interconversion (156). The structural models are based on the structures of the cytoplasmic state of the bovine ADP/ATP carrier (PDB:1OKC) (128) and the matrix state of the ADP/ATP carrier of *Thermothelomyces thermophila* (PDB:6GCI) (142). The black spheres with roman numerals are the contact points of the substrate binding site (94, 136). IS, intermembrane space; IM, inner membrane; MM, mitochondrial matrix.

Mitochondrial carriers consist of three homologous repeats of ~100 amino acid residues each (FIGURE 3A) (147). The three repeats fold up into a threefold pseudo-symmetrical fold, noted first in the projection structure of the yeast ADP/ATP carrier Aac3p (92). This study also demonstrated that the carrier had a monomeric structure and a translocation path for substrates through the center of the protein (92). The first atomic structure of the bovine ADP/ATP carrier provided evidence for the basic structural topology of all mitochondrial carriers (128). Each repeat or domain has an odd-numbered helix (H1, H3, H5), a matrix loop of variable length, a matrix helix (h12, h34, h56), a linker helix (l12, l34, l56), and an even-numbered helix (H2, H4, H6) (128) (FIGURE 3, A AND B). The domains are linked by cytoplasmic loops, which are located in the intermembrane space, together with the NH_2_ and COOH termini. The structure was locked by the specific inhibitor carboxyatractyloside in the cytoplasmic state in which the central cavity is open to the intermembrane space for binding of ADP (FIGURES 4 AND 5) (128). Although there are some structural differences, the same basic structural fold was observed for the yeast ADP/ATP carrier Aac2p and Aac3p, even though they share only ~50% identity with the bovine carrier (141). More recently, the atomic structure of the mitochondrial ADP/ATP carrier inhibited by bongkrekic acid has been solved, locked in the matrix state in which the central cavity is open to the mitochondrial matrix, for binding of ATP (FIGURES 4 AND 5) (142, 144). The central cavity in both states is positively charged, primed for binding of the negatively charged adenine nucleotides (128, 142, 144). The residues of the substrate binding site have been identified by computational methods, using chemical and distance constraints in comparative models (94, 136), deviation of symmetry (95, 137), or molecular dynamics simulations (35, 107, 166). The three main contact points involved in substrate binding are located on the even-numbered helices (FIGURES 4 AND 5) (94, 136). The substrate binding site is located at the bottom of a water-filled cavity in both states, which corresponds to the middle of the membrane (94, 136). There are two gates on either side of the carrier that regulate access to the central binding site. In the cytoplasmic state, the matrix gate is closed and the cytoplasmic gate is open, whereas, in the matrix state, the cytoplasmic gate is closed and the matrix gate is open (FIGURES 4 AND 5) (142–144). Each closed gate is ~15-Å thick, an important insulation layer to prevent the leak of protons and other ions in the presence of a 180-mV membrane potential. These gates contain salt-bridge networks of positively and negatively charged amino acid residues and other features, which will be discussed later.

### Transport Mechanism of Mitochondrial Carriers

Comparison of the two inhibited states has revealed the basic structural mechanism of transport and has completed our understanding of the importance of key conserved sequence features of the SLC25 family for the transport mechanism (FIGURE 5) (142–144). The two inhibitors occupy the proposed substrate binding site of the carrier, preventing substrate binding, but also induce slight conformational changes, which lock the carrier permanently in an abortive state (142). These distortions can be corrected for structurally to achieve a closer approximation of the unliganded states, which are used in this review. A morph between the unliganded states passes through an occluded state, where access to the substrate binding site is blocked from both sides of the membrane, which is a requirement for an alternating access transport mechanism (142).

Further analysis shows that most of the domain structure is conserved between the two states, i.e., the odd-numbered helix, the matrix helix, the linker helix and a third of the even-numbered helix (142–144). These parts of the domain are called the core elements and are shown in primary colors blue, yellow, and red for the first, second, and third domain, respectively (FIGURE 5). In contrast, a significant change occurs in the position of the COOH-terminal regions of the even-numbered helices in the state interconversion (143, 144). These parts of the structure are called the gate elements, and they are shown in gray (FIGURE 5). The hinge points of these movements turned out to be the contact points of the proposed substrate binding site (142–144), identified previously (94, 136). Opening or closing of the matrix side of the carrier involves the rotation of the three core elements as rigid bodies, whereas opening or closing of the cytoplasmic side requires the rotation of the three gate elements. In this way, access to the central substrate binding site from one or the other side of the membrane is alternated (FIGURE 5). The substrate binding site is the fulcrum of these movements (142–144). Thus state interconversion requires the coordinated movement of six elements simultaneously, making the mitochondrial carriers one of the most dynamic of all transport proteins. These movements are facilitated by the transmembrane helices being held together only by relatively weak van der Waals interactions. Next, the sequence features of mitochondrial carriers are examined to determine why they are crucial to the structure and transport mechanism, since they are often altered in pathogenic variants.

### Key Sequence Features Affected by Pathogenic Mutations

The easiest way to present the sequence features of mitochondrial carriers is to use their threefold pseudo-symmetry (FIGURE 3). The three repeats are homologous to each other, meaning that residues that are in the same position in each repeat are symmetry-related and thus mostly identical or similar in physiochemical properties (137). These residues can be grouped together in a triplet of symmetry-related residues, which facilitates their comparison (FIGURE 3A). Here, we have used the residue numbering of the human ADP/ATP carrier 1 (AAC1, SLC25A4), also known as ANT1, to relate the triplet to the original sequence. For example, the highlighted triplet (cyan sphere) in FIGURE 3A contains *residue 8* in *repeat 1, residue 113* in *repeat 2*, and *residue 210* in *repeat 3* (FIGURE 3). For convenience, the entire triplet is named by the residue number in the first repeat in SLC25A4, meaning that the example above would be called *triplet 8*. Given the high sequence identity within the SLC25 family, the same triplets in carriers with different functions can be compared to look for ones that are conserved throughout the family and are therefore universally important for the structure and mechanism of mitochondrial carriers (137).

**Figure 6:**
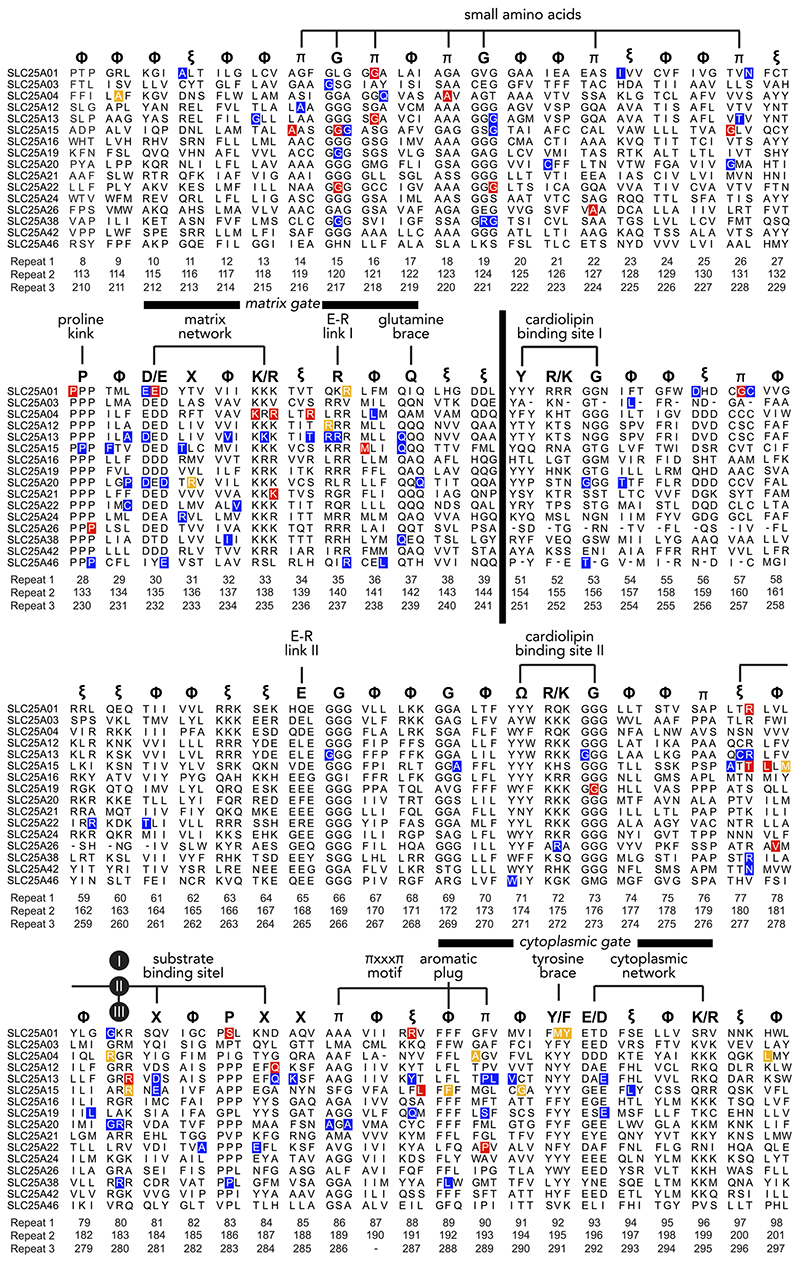
Pathogenic mutations observed in disease variants of mitochondrial carriers Aligned triplets of the 16 mitochondrial carriers associated with developmental, metabolic, and neuromuscular diseases. The mutations that have a severe effect on function are shown in red boxes, whereas those that have milder effects are in yellow. Mutations in blue boxes have been identified by genetic analysis, but their effect has not been studied experimentally. At the *bottom* are the three residue numbers that form a triplet in the human ADP/ATP carrier (SLC25A4). At the *top* are the conserved structural and functional features of mitochondrial carriers. The triplet is labeled by the one-letter code of the most conserved residue or by the most common property: π, small amino acids; Φ, hydrophobic amino acids;ξ, hydrophilic amino acids; Ω, aromatic amino acids; or by X for any amino acid. The black spheres with roman numerals are the contact points of the substrate binding site (94, 136). H6 in the ADP/ATP carrier is one residue shorter than other carriers and lacks a residue in triplet 90. The matrix loops (indicted by the black bar), as well as the cytoplasmic loops and NH_2_ and COOH terminus have been omitted.

FIGURE 6 shows the triplets of all conserved helical features for the 16 mitochondrial carriers that have been linked to human disease, as well as the reported pathogenic mutations. The mutations that have been experimentally verified to have a severe effect on function are shown in red boxes, whereas those that have milder effects are in yellow ones. Mutations shown in blue boxes have been flagged in genetic analysis, but their effect on function has not been studied experimentally. It is clear that mutations affect a large number of triplets. Next, starting from the NH_2_-terminus, these features will be presented by highlighting the properties of the triplets in relation to the known structural mechanism of transport (FIGURE 6). The analysis focuses on the conserved helical parts of the carriers, since the loops are highly variable in length and sequence, and do not play an important role in the transport mechanism. The most conserved amino acid residues of the triplets are indicated by the one-letter amino acid code, whereas the most common chemical and physical properties are also indicated by a one-letter code: π for small residues (Gly, Ala, Ser, Pro, Cys, Val, Thr), Φ for hydrophobic residues (Val, Ile, Leu, Phe, Trp, Tyr, Cys, Ala, and Met), Ω for aromatic residues (Trp, Tyr, Phe), ξ for hydrophilic residues (Asn, Gln, Glu, Asp, Lys, Arg, His, Ser, Thr), and X for any residues.

### Small Amino Acid Residues on the Odd-Numbered Helices

**Figure 7:**
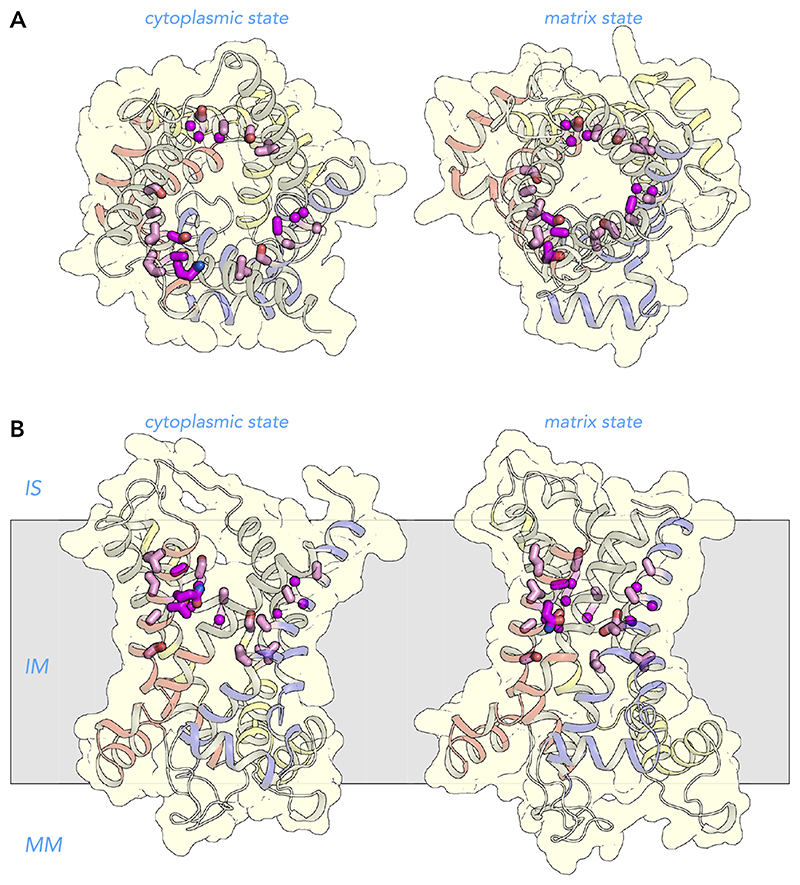
Small amino acid residues on the odd-numbered helices View from the intermembrane space (*A*) and lateral view from the membrane (*B*) of the human ADP/ATP carrier in the cytoplasmic state (*left*) and the matrix state (*right*). The structural models are based on the structures of the cytoplasmic state of the bovine ADP/ATP carrier (128) and the matrix state of the ADP/ATP carrier of *Thermothelomyces thermophila* (142). The carrier is shown in surface representation and the helices in cartoon representation with the core elements in primary colors and the gate elements in gray. Glycine or small residues in the interface with the preceding helix are shown in pink, whereas those in the interface with the following helix are shown in magenta. The sequence motif is πGπxπGxxπxxxπ, where G stands for glycine and π for small amino acids (142–144). When the carrier changes from the cytoplasmic state to the matrix state, the inter-helical distances become smaller on the cytoplasmic side of the carrier to facilitate the formation of the cytoplasmic network, requiring small residues in the helical interfaces. IS, intermembrane space; IM, inner membrane; MM, mitochondrial matrix.

The odd-numbered helices H1, H3, and H5 have a strong kink, giving them an L shape (FIGURES 3 AND 4) (128). The NH_2_-terminal parts are transmembrane and contain a large number of glycine and other small residues, which are often mutated in disease variants of the carriers (FIGURE 6). The extended sequence motif is πGπxπGxxπxxxπ, where G stands for glycine, π for small amino acids, and x for any amino acid residue (142–144) (FIGURE 7). These residues can be divided further into two categories: *1*) small residues in the interface with the preceding helix, i.e., *triplets 14, 18, 22*, and *26* (pink residues, FIGURE 7), and *2*) glycine or small residues in the interface with the following helix, i.e., *triplets 15* and *19*, which form the GxxxG motif, and *triplet 16* (magenta residues, FIGURE 7) (141–144).

When the carrier transitions from the cytoplasmic state to the matrix state (FIGURES 4 AND 5), the cytoplasmic side of the carrier closes and the transmembrane helices come together. The reason is that the cytoplasmic side closes because the gate elements rotate inward, allowing the cytoplasmic network to form (see below). At the same time, the core elements rotate outward, and simultaneously the odd helices move inward on the cytoplasmic side. The glycine and other small residues of the πGπxπGxxπxxxπ motif are in these crucial interhelical interfaces, allowing movement of the gate elements across the surface of the odd-numbered helices and the close proximity of the helices in the matrix state. A large number of pathogenic mutations are observed in these triplets, and functional and structural analysis shows that these residues are likely to be important for the mechanism of mitochondrial carriers in general (25, 33, 90). Small residues can also be found on the even-numbered helices, as will be discussed below.

The other triplets found on the transmembrane parts of the odd-numbered helices preserve their strong amphipathic properties. *Triplets 8, 9, 10, 12, 13, 17, 20, 21, 24*, and *25* contain mostly generic hydrophobic residues (Φ symbols, FIGURE 6), since they point toward the hydrophobic core of the membrane. *Triplets 11, 23*, and *27* contain generic hydrophilic residues (ξ symbols, FIGURE 6) and point toward the water-filled cavity. Pathogenic mutations are sporadically observed in these triplets, most likely when they are replaced with a residue with the opposite properties. For instance, a charged residue for a hydrophobic one, a large residue for a small one, or a substitution with a proline residue in the middle of a helix, which could break the helix. These features could be important for both the function and biogenesis of the carriers.

### Key Amino Acid Residues of the Matrix Gate

**Figure 8:**
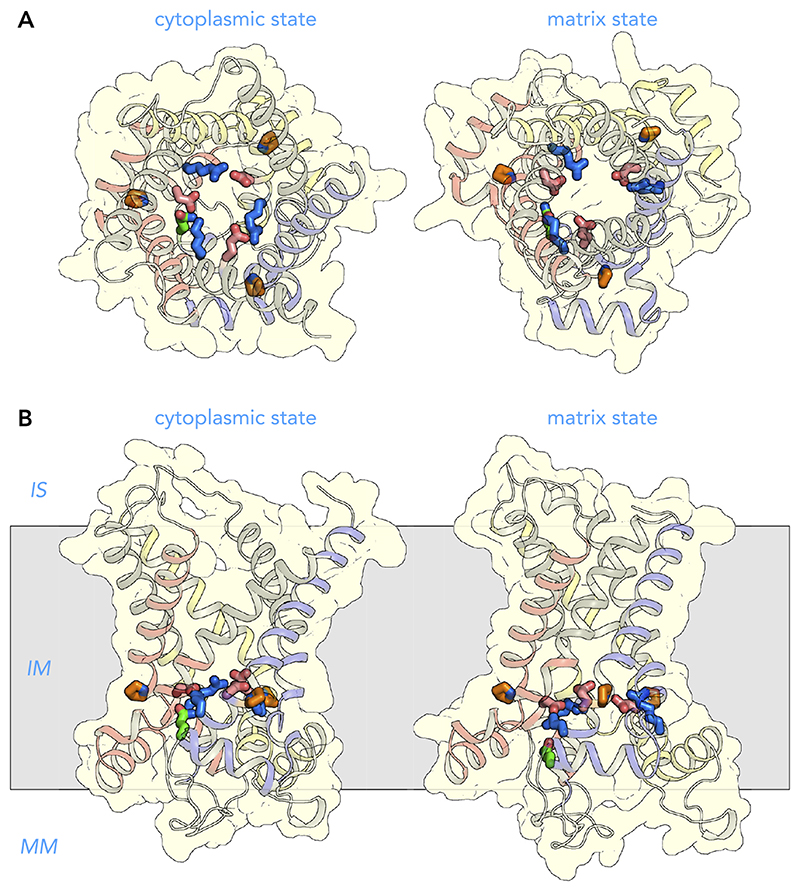
Key amino acid residues of the matrix gate View from the intermembrane space (*A*) and lateral view from the membrane (*B*) of the human ADP/ATP carrier in the cytoplasmic state (*left*) and matrix state (*right*). The structures are described in the legend to FIGURE 7. The key residues shown belong to the sequence motif Px[DE]xx[RK]xxxQ on the odd-numbered helices. The proline residues (orange) are found at the pronounced kink in the odd-numbered helices, bringing the negatively charged (red) and positively charged (blue) residues together to form the matrix network in the cytoplasmic state. Underneath one of the salt bridges is a glutamine residue (green) that forms hydrogen bonds with both residues (glutamine brace), but in other carriers one, two, or three glutamine braces can be found (FIGURE 6) (141). IS, intermembrane space; IM, inner membrane; MM, mitochondrial matrix.

The next important motif on the odd-numbered helices is a highly conserved symmetrical feature Px[DE]xx[RK]xxxQ (FIGURE 6). As mentioned above, the odd-numbered helices have a strong kink of ~50 degrees (FIGURE 5). At this kink is a highly conserved proline residue (P-kink, *triplet 28*), the first residue of the motif (128), but it can be replaced by a serine residue (141). These residues break the hydrogen bond arrangement, allowing the kink to occur, and a network of interactions between residues in the domain help to stabilize it (141) (FIGURE 8). The kinks bring the COOH-terminal ends of the odd-numbered helices together in the center of carrier in the cytoplasmic state, where the negatively charged residues (red residues, *triplet 30*) and positively charged residues (blue residues, *triplet 33*) form an ionic interaction network (117). This interaction network can be seen in the structure of the cytoplasmic state (128, 141), now called the matrix salt-bridge network (137). A glutamine residue (*triplet 37*) functions as a brace of one of the salt-bridge interactions of the matrix network (glutamine-brace) (green residue, FIGURE 8) (141). Only one of the three domains of the ADP/ATP carrier has a glutamine brace, but other carriers have up to three glutamine braces (FIGURE 6). The salt-bridge interactions and glutamine braces together determine the overall interaction energy of the matrix network. Although not conserved between different carriers, residues in *triplets 34* and *38* are in the translocation path, sealing the carrier to the mitochondrial matrix (FIGURE 6). Together, all these residues form the matrix gate, which is closed in the cytoplasmic state and open in the matrix state (FIGURES 5 AND 6). In addition, there are triplets with hydrophobic residues (*triplet 29*, *32*, *36*), hydrophilic residues (*triplet 34, 38*), and residues with varied properties (*triplet 31*). This part of the carrier is one of the most important for their function, and a large number of pathogenic variants have been identified in this region (FIGURE 6).

### Amino Acid Residues Involved in Cardiolipin Binding

**Figure 9:**
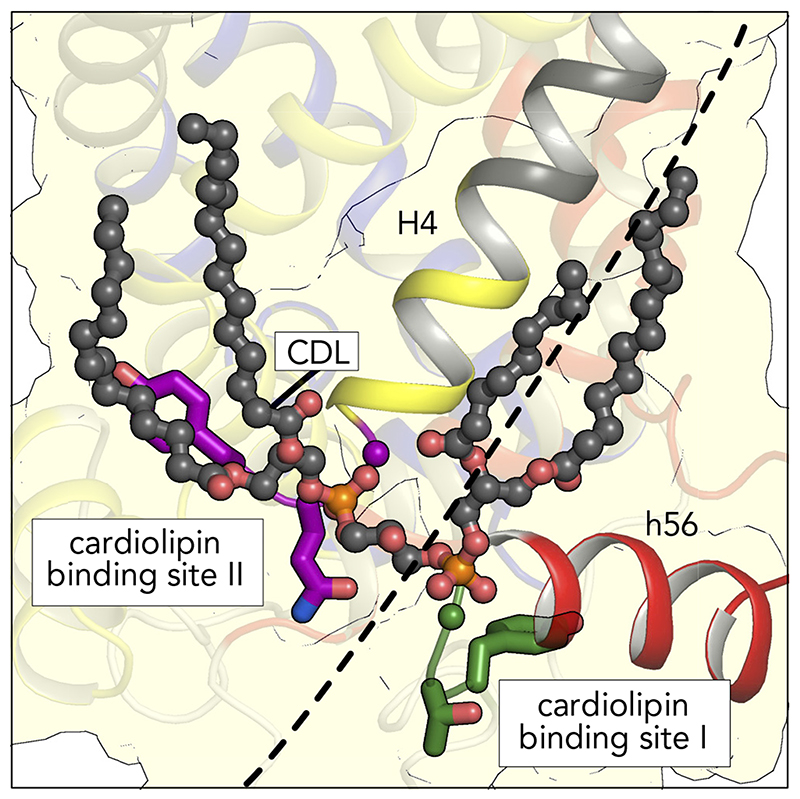
Detailed view of one of the three binding sites for cardiolipin One cardiolipin molecule is shown in ball-and-stick representation (cdl802, PDB: 2C3E), which is bound on the surface of the carrier and spans the inter-domain interface. The carrier is shown in cartoon representation with transmembrane H4 (yellow and gray) and matrix helix h56 (red) enhanced. The two phosphate groups of cardiolipin, which are linked by a glycerol moiety, form hydrogen bonds with the amide groups (128) and interact with the positively charged ends of the helix dipoles of the NH_2_-terminal ends of the matrix helices (cardiolipin binding site I) and the even-numbered helices (cardiolipin binding site II) (24, 141, 142). The four fatty acid chains of cardiolipin interact with the surface of the carrier in a non-specific way via van der Waals interactions (41). Residues in the conserved cardiolipin binding site I and II are shown as green and purple sticks, respectively. The interface between *domain 2* and *3* is shown as a dashed line.

**Figure 10:**
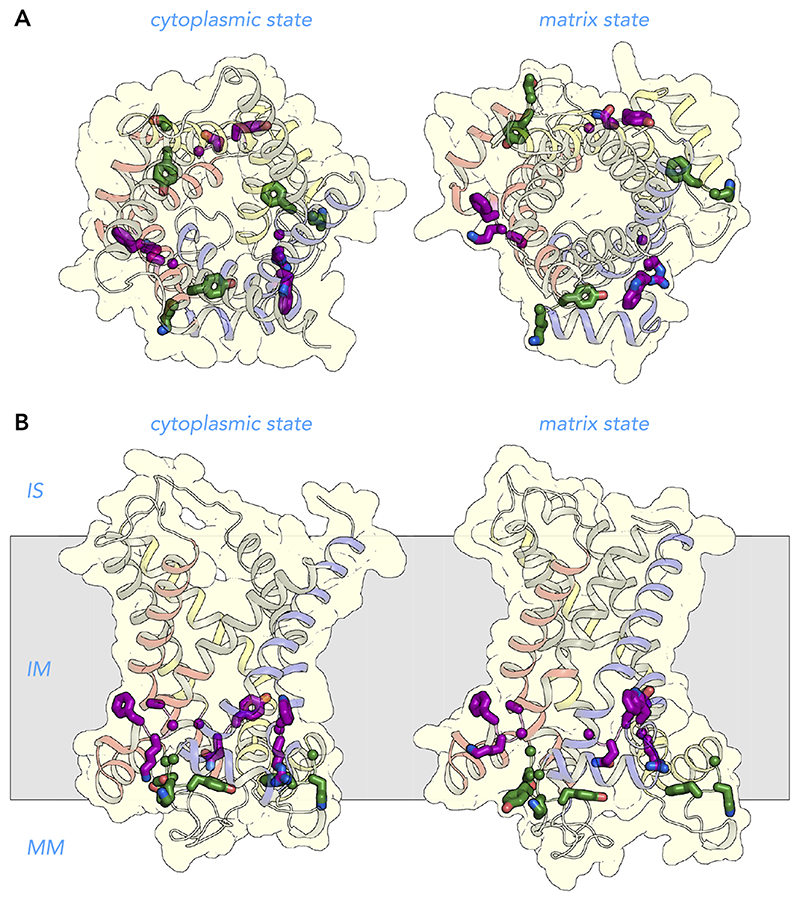
Amino acid residues involved in cardiolipin binding View from the intermembrane space (*A*) and lateral view from the membrane (*B*) of the human ADP/ATP carrier in the cytoplasmic state (*left*) and matrix state (*right*). The structures are described in the legend to FIGURE 7. There are three highly conserved binding sites for cardiolipin in the mitochondrial ADP/ATP carrier. The residues of cardiolipin binding site I belong to the [YF]xG motif (green), whereas those of cardiolipin binding site II belong to the [YWF][RK]G motif (purple) (128, 141, 142). IS, intermembrane space; IM, inner membrane; MM, mitochondrial matrix.

Three cardiolipin molecules are tightly bound to mitochondrial carriers and are important for their stability and function, as observed by phosphorous NMR experiments (14), crystallographic analyses (128, 141, 142), lipid analysis (10, 93, 96), disease models (56, 74, 104), thermostability analysis (34), and transport assays (63, 75, 107). The phosphate groups of cardiolipin are bonded to the NH_2_-terminal ends of the matrix and even-numbered helices, bridging the inter-domain interface (FIGURE 9). Preceding them are highly conserved symmetrical sequence motifs [YF]XG (*triplets 51–53*, cardiolipin binding site I) and [YWF][RK]G (*triplets 71–73*, cardiolipin binding site II), respectively (FIGURES 6 AND 10). The glycine residues of these motifs (*triplets 53* and *73*) are in the loop to helix transition, where they function as helix breakers, but serine, asparagine, or threonine can also play this role (24). This loop-to-helix transition is crucial for binding of the phosphate groups of cardiolipin via hydrogen bonds (128) and electrostatic interactions with the helix dipoles (141). Many pathogenic mutations affect these glycine residues (FIGURE 6). The aromatic residue of cardiolipin binding site I (*triplet 51*) is involved in stabilization of the domain on the matrix side (108), whereas the aromatic residue of cardiolipin binding site II (*triplet 71*) is involved in binding the fork of the lipid moiety (128, 141, 142). Although not supported by the structures, molecular dynamics simulations show that the positive charged residue (*triplet 72*) might be involved in binding the phosphate moiety via electrostatic interactions (41). *Triplet 71–72* have pathogenic mutations, supporting their importance in stability and function of the carriers.

### Amino Acid Residues Important for the Stability of the Domain Structure

**Figure 11:**
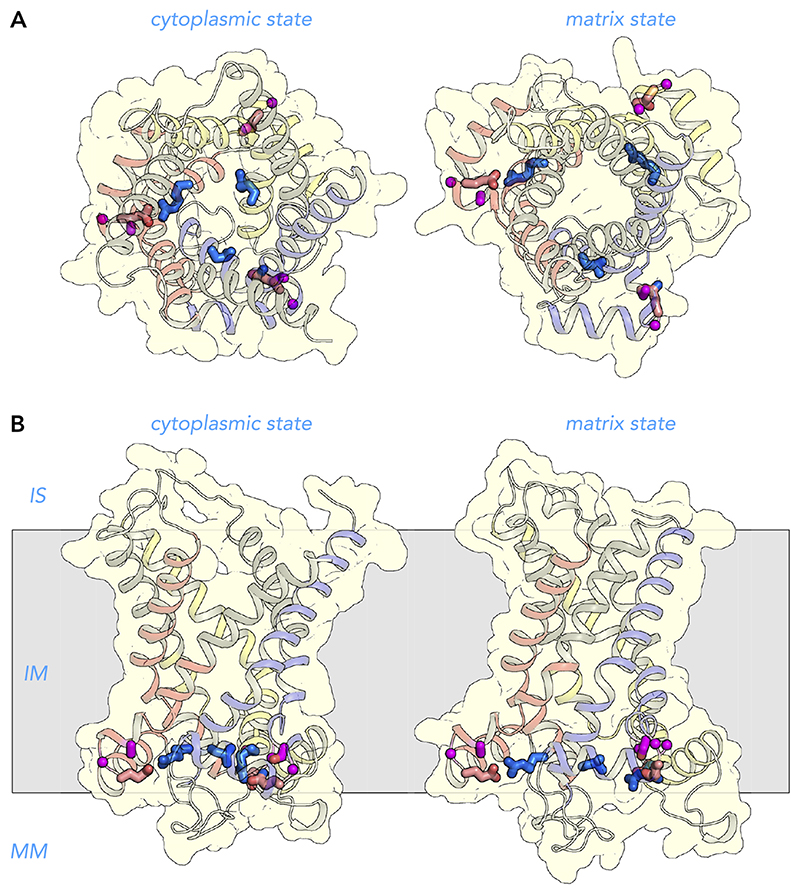
Amino acid residues important for the domain structures on the matrix side View from the intermembrane space (*A*) and lateral view (*B*) from the membrane of the human ADP/ATP carrier in the cytoplasmic state (left) and matrix state (*right*). The structures are described in the legend to FIGURE 7. The positively charged residue of the E-R link I (blue), which is located in the matrix gate area, interacts with the negatively charged residue of the E-R link II, which is located on the matrix helices. The NH_2_-terminal and the fourth residues of the linker helices are most commonly glycine residues (magenta), although the latter can be replaced by other small residues. IS, intermembrane space; IM, inner membrane; MM, mitochondrial matrix.

Analysis of all of the polar interactions of the ADP/ATP carrier show that there are no conserved polar interactions between the transmembrane helices (141), which agrees with a mechanism where the transmembrane helices move relative to each other in the state interconversion (FIGURE 5) (142). The only highly conserved interaction is in the domain structure between a positively charged residue on the odd-numbered helices (E-R link I, *triplet 35*) and a negatively charged residue on the matrix helices (E-R link II, *triplet 65*) (FIGURES 6 AND 11). In the known structures of the ADP/ATP carrier, one or two interactions are evident, supporting the unusual shape of the domain structure (128, 141, 142). One of the residues of E-R link I in repeat I is a leucine, which cannot be involved in a direct interaction with the glutamine residue in E-R link II (FIGURE 6). However, a preceding residue, Arg31, which is located one turn of a helix away, is in bonding distance, thus fulfilling the same role (FIGURE 6). On the basis of sequence analysis, it is likely that all three domains have this interaction in other carriers, which it has been shown to be important for function (108).

Another residue important for the stability is a tyrosine residue in cardiolipin binding site I (*triplet 51*), which forms extra interactions and seals the domain toward the mitochondrial matrix (FIGURE 10). The E-R link often contains pathogenic mutations, further supporting its importance.

Residues of the matrix helices are strongly amphipathic, with polar and charged residues facing the mitochondrial matrix (*triplets 56, 59, 60, 63, 64*) and hydrophobic residues facing the membrane (*triplets 54, 55, 58, 61, 62*) (FIGURE 6). Small residues are also required for the stability of the domain structure as helix breakers, such as glycine residues (*triplet 53, 66, 73*) or small residues pointing toward other residues (*triplet 57*, *69*) (FIGURE 11). These areas are affected by pathogenic mutations, likely because the mutations cause changes to amino acids with opposite biophysical properties.

### Amino Acid Residues of the Substrate Binding Site

**Figure 12:**
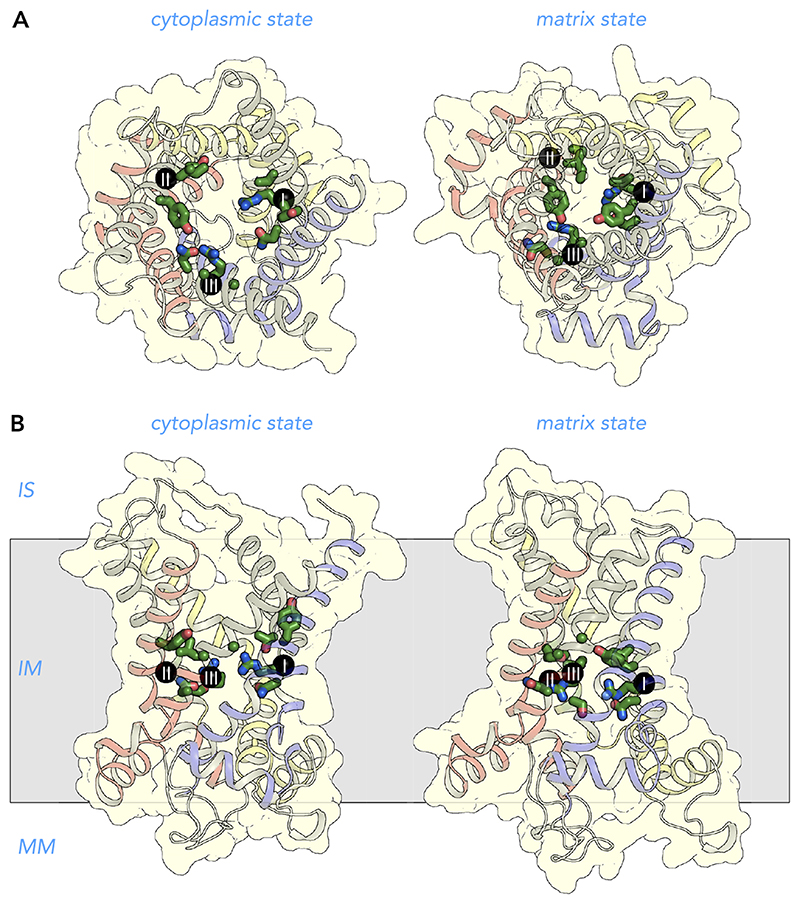
Amino acid residues of the substrate binding site View from the intermembrane space (*A*) and lateral view (*B*) from the membrane of the human ADP/ATP carrier in the cytoplasmic state (*left*) and matrix state (*right*). The structures are described in the legend to FIGURE 7. The black spheres with roman numerals are the contact points of the substrate binding site, which are involved in binding of the substrates (94, 136). Other residues in this site (green) may either bind substrate directly or may allow the binding. IS, intermembrane space; IM, inner membrane; MM, mitochondrial matrix.

A single substrate binding site can be found in the central cavity, approximately in the middle of the membrane. The binding site consists of residues that are directly involved in binding of the substrate, such as the contact points (FIGURES 4, 5, AND 12), but also of residues that allow the binding of the substrate in the water-filled cavity. This area is a hyper-variable region and contains a large number of asymmetric residues (137). Most of these residues can be found on the even-numbered helices at *triplets 77, 80* (contact points), *81*, *84*, and possibly *85*. There are many disease variants that have mutations in this area and that have been shown to affect function in functional studies. There are also triplets that often contain proline residues, such as *triplets 76* and *83*, which can be found on either side of the contact points (*triplet 80*) and may facilitate the curvature or the relative movement of the helices.

### Small Amino Acid Residues on the Even-Numbered Helices

**Figure 13:**
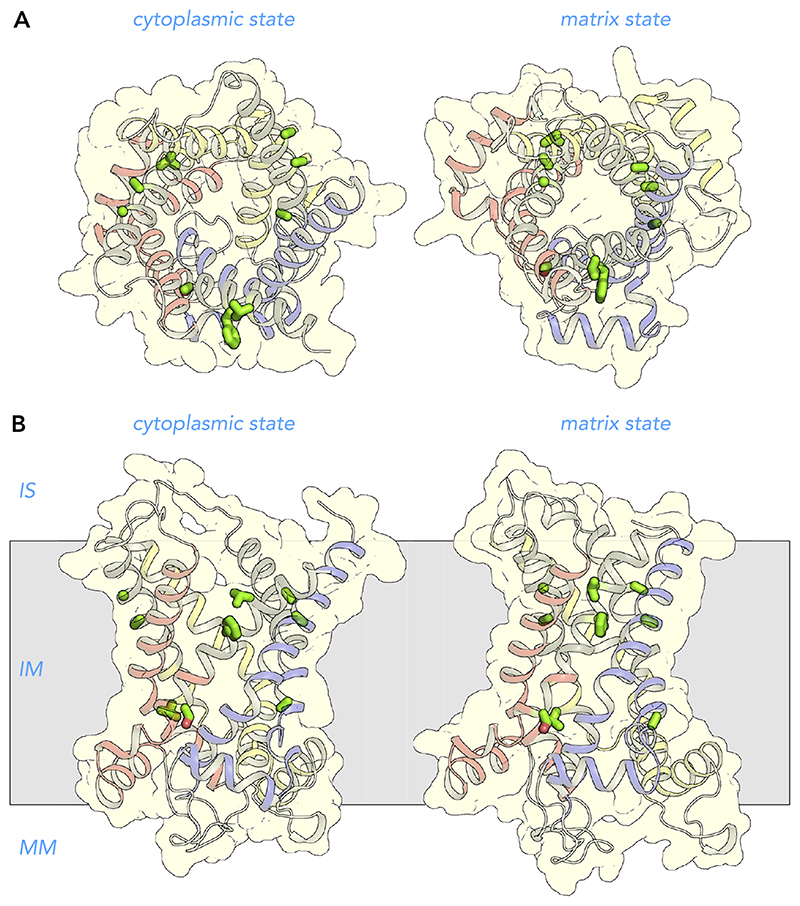
Small amino acid residues on the even-numbered helices View from the intermembrane space (*A*) and lateral view (*B*) from the membrane of the human ADP/ATP carrier in the cytoplasmic state (*left*) and matrix state (*right*). The structures are described in the legend to FIGURE 7. The small residues (chartreuse) in the interhelical interfaces with the odd-number helices facilitate conformational changes. Some residues are larger, such as the phenylalanine on H6, because their side chains face the membrane in both conformations. IS, intermembrane space; IM, inner membrane; MM, mitochondrial matrix.

As explained earlier, the state interconversion requires the presence of small residues in the interhelical interfaces. Several can be found on the even-numbered helices that facilitate these movements, such as the aforementioned *triplet 76*, and a πxxxπ motif, formed by *triplets 86* and *90* (FIGURE 6 AND 13) (142). The even-numbered helices are also highly amphipathic, containing hydrophobic residues in *triplets 74*, *75*, *78*, *79*, *82*, *87*, and *91*, facing the membrane, and hydrophilic residues facing the cavity, such as the substrate binding site residues, and *triplets 88, 94*, and *97* (FIGURE 6).

### Amino Acid Residues of the Cytoplasmic Gate

**Figure 14:**
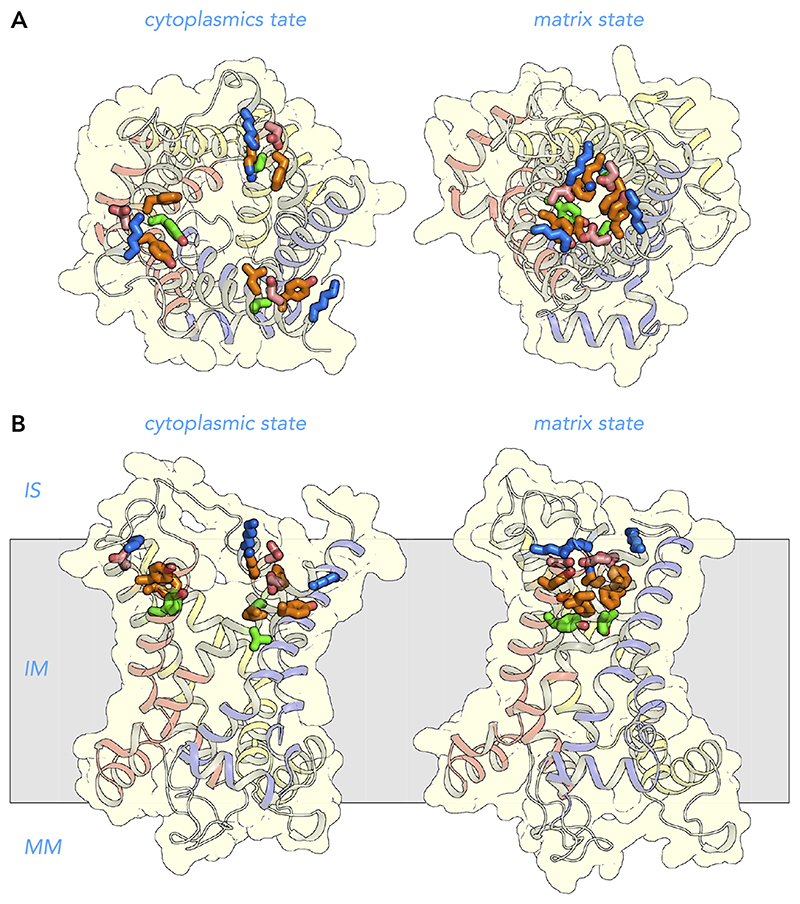
Amino acid residues of the cytoplasmic gate View from the intermembrane space (*A*) and lateral view (*B*) from the membrane of the human ADP/ATP carrier in the cytoplasmic state (*left*) and matrix state (*right*). The structures are described in the legend to FIGURE 7. The key residues belong to the sequence motif ξ[FY]xx[YF][DE]xx[RK]. The negatively charged (red) and positively charged (blue) residues together form the cytoplasmic network in the matrix state. Underneath are aromatic residues (orange), which are part of the hydrophobic plug that closes the cytoplasmic gate. The aromatic residue preceding the negatively charged residue is the tyrosine brace (Y-brace) (142–144). Preceding the aromatic residues are hydrophilic residues (ξ), which form the ceiling of the substrate binding site. IS, intermembrane space; IM, inner membrane; MM, mitochondrial matrix.

The COOH-terminal ends of the even-numbered helices contain highly conserved symmetrical sequence motifs ξ[FY]xx[YF][DE]xx[RK] (*triplets 88–96*). Together, they form the cytoplasmic gate (FIGURE 6). The negatively charged residues (*triplet 93*) and positively charged residues (*triplet 96*) form the cytoplasmic salt-bridge network when the carrier is in the matrix state (80, 142, 144), whereas this network is disrupted in the cytoplasmic state (red and blue residues, FIGURE 14) (141). The preceding aromatic residue (*triplet 92*), most often a tyrosine residue, can form hydrogen-bond interactions with the negatively charged residue of the neighboring domain (orange residue, FIGURE 14). This interaction is called the tyrosine brace, and mitochondrial carriers have one to three of these interactions, modifying the overall interaction energy of this network. Together with *triplet 89*, it also doubles up as a hydrophobic layer when the carrier is in the matrix state. *Triplet 88*, which is quite variable, is part of the cytoplasmic gate and also forms part of the ceiling of the substrate binding site (green residues, FIGURE 14). These residues are often mutated in disease variants.

## Concluding Remarks

Mitochondrial carriers are highly dynamic transporters, which interconvert between a cytoplasmic state and a matrix state by using six dynamic elements, comprising three core elements and three gate elements. They are among the smallest transporters in nature, yet they transport some of the largest molecules, such as adenine nucleotides, *S*-adenosyl methionine, flavins, and acyl-carnitines. They do so without significant proton leak, because of a matrix and cytoplasmic gate, both with salt-bridge networks and braces, and other residues that provide an insulation layer. All carriers have a single central substrate binding site with three contact points, which is alternately accessible from one side of the membrane or the other, key properties of an alternating access transport mechanism. A large number of pathogenic mutations have been identified, which cause a range of metabolic, neuromuscular, and developmental diseases. The vast majority of them can be explained because they affect key structural and functional features of mitochondrial carriers. Some of them are found in loop regions, which have not been included in this analysis, and others in extra domains, such as the regulatory domain of the aspartate/glutamate carrier, which have been explained before (FIGURE 2C) (157). The few that are remaining often involve mutations that introduce different properties from those of the original residue (e.g., p.Asp69Tyr in SLC25A1, p.Cys23Arg in SLC25A20, or p.Thr56Pro in SLC25A22), which could impair the structure and function but also the biogenesis of the carrier. To understand these diseases, it is really important to discriminate between these different options, but the majority of these pathogenic mutations have not been studied experimentally. Given the fact that so many amino acid residues are important for the function of mitochondrial carriers and that the SLC25 family is the largest solute carrier family in humans, it is likely that many more disease variants will be discovered.
